# Implications of Measles Inclusion by Commercial Syndromic Polymerase Chain Reaction Panels — United States, May 2022–April 2023

**DOI:** 10.15585/mmwr.mm7312a3

**Published:** 2024-03-28

**Authors:** Christine M. Thomas, Amanda Hartley, Ann Schmitz, Heather D. Reid, Susan Sullivan, Elise Huebner, Meredith Robinson, Adria Mathis, Mary-Margaret A. Fill, Kara J. Levinson, Tim F. Jones, William Schaffner, Caitlin N. Newhouse, John R. Dunn

**Affiliations:** ^1^Tennessee Department of Health; ^2^Epidemic Intelligence Service, CDC; ^3^Florida Department of Health; ^4^Career Epidemiology Field Officer Program, CDC; ^5^Illinois Department of Public Health; ^6^North Carolina Department of Health and Human Services; ^7^Texas Department of State Health Services; ^8^Virginia Department of Health; ^9^Division of Viral Diseases, National Center for Immunization and Respiratory Diseases, CDC; ^10^Division of Laboratory Services, Tennessee Department of Health; ^11^Vanderbilt University Medical Center, Nashville Tennessee.

**Summary**

**What is already known about this topic?**

Syndromic polymerase chain reaction panels test for pathogens that can cause rash illnesses, including measles. Rash illnesses have many causes. Approximately 5% of patients experience a rash after receipt of a measles, mumps, and rubella vaccine.

**What is added by this report?**

Among syndromic panels conducted by a commercial laboratory, approximately 1% were positive for measles. Patients who received these results were children without known measles risk factors who had been vaccinated against measles, the majority <3 weeks previously. Their results were attributed to detection of measles vaccine virus.

**What are the implications for public health practice?**

After vaccination, syndromic panels can detect measles vaccine virus, which is not transmitted to others and does not cause disease in immunocompetent persons. Any detection of measles virus should be immediately reported to public health agencies to determine appropriate public health response.

## Abstract

Syndromic polymerase chain reaction (PCR) panels are used to test for pathogens that can cause rash illnesses, including measles. Rash illnesses have infectious and noninfectious causes, and approximately 5% of persons experience a rash 7–10 days after receipt of a measles, mumps, and rubella (MMR) vaccine. MMR vaccine includes live attenuated measles virus, which is detectable by PCR tests. No evidence exists of person-to-person transmission of measles vaccine virus, and illness does not typically result among immunocompetent persons. During September 2022–January 2023, the Tennessee Department of Health received two reports of measles detected by syndromic PCR panels. Both reports involved children (aged 1 and 6 years) without known risk factors for measles, who were evaluated for rash that occurred 11–13 days after routine MMR vaccination. After public health responses in Tennessee determined that both PCR panels had detected measles vaccine virus, six state health departments collaborated to assess the frequency and characteristics of persons receiving a positive measles PCR panel test result in the United States. Information was retrospectively collected from a commercial laboratory testing for measles in syndromic multiplex PCR panels. During May 2022–April 2023, among 1,548 syndromic PCR panels, 17 (1.1%) returned positive test results for measles virus. Among 14 persons who received a positive test result and for whom vaccination and case investigation information were available, all had received MMR vaccine a median of 12 days before specimen collection, and none had known risk factors for acquiring measles. All positive PCR results were attributed to detection of measles vaccine virus. Increased awareness among health care providers about potential measles detection by PCR after vaccination is needed. Any detection of measles virus by syndromic PCR testing should be immediately reported to public health agencies, which can use measles vaccination history and assessment of risk factors to determine the appropriate public health response. If a person recently received MMR vaccine and has no risk factors for acquiring measles, additional public health response is likely unnecessary.

## Introduction

Syndromic polymerase chain reaction (PCR) panels are used to test for pathogens that can cause rash illnesses, including measles. Rash illnesses have infectious and noninfectious causes, and approximately 5% of persons experience a rash 7–10 days after receipt of a measles, mumps, and rubella (MMR) vaccine (*1*). A component of MMR vaccines is live attenuated measles virus (genotype A). Although measles vaccine virus is detectable by PCR tests that target the nucleocapsid gene, no evidence of person-to-person transmission exists (*2**,**3*). Measles vaccine virus does not cause measles infection and does not typically cause illness among immunocompetent persons (*1**,**4*).

During September 2022–January 2023, the Tennessee Department of Health received two reports of measles in children aged 1 and 6 years, who were evaluated for rash illnesses without documented exposures or plausible epidemiologic risk factors for measles. Rashes occurred 11–13 days after administration of their routine first dose of MMR vaccine.^†^ Both children received a positive measles test result on syndromic PCR panels for rash illnesses, a platform that simultaneously tests for multiple pathogens. Public health investigations of both reports concluded that positive test results represented detection of the live attenuated measles virus used in MMR vaccine. 

Syndromic PCR platforms that report positive measles test results in persons without measles infection could result in extensive, unnecessary public health responses to measles vaccine virus detected after MMR vaccination. To guide future public health response to syndromic PCR panel detection of measles virus, six state health departments^§^ assessed the frequency and characteristics of persons receiving positive measles PCR panel test results in the United States since a commercial platform became available in May 2022.^¶^

## Methods

A commercial laboratory that included measles testing as part of a syndromic multiplex PCR panel provided the numbers of such panels ordered in the United States during May 2022–April 2023 and the number that detected measles virus by state. Six state health departments in states where syndromic PCR panels had detected measles virus partnered to collect patient information from laboratory reports, including age, sex, location and date of specimen collection, and whether other pathogens were detected. State immunization information systems were queried to identify patient MMR vaccination status and date of vaccination. Clinicians who ordered tests were asked about the patients’ clinical signs and symptoms** and epidemiologic risk factors for measles (i.e., recent international travel or known exposure to a person with measles virus infection). For the initial two test results reported in Tennessee, findings were collected from subsequent measles testing, including a measles vaccine assay (MeVA) or viral genotype^††^ (*2**,**5**,**6*). This activity was reviewed by CDC, deemed not research, and was conducted consistent with applicable federal law and CDC policy.^§§^

## Results

### Detection of Measles Virus by Syndromic PCR Panels

During May 2022–April 2023, clinicians nationwide ordered 1,548 syndromic PCR panels from the participating commercial laboratory. In the 25 states where these clinicians practiced, a median of 13 tests were ordered per state (range = 1–368 tests). Among all tests conducted, 17 (1.1%) detected measles virus (Figure). Tennessee clinicians ordered the most tests (368) and detected measles most frequently (seven; 1.9%). Laboratory reports were available for 14 (82.4%) of 17 syndromic PCR panels that detected measles; these tests were ordered at pediatric (10), urgent care (three), and family practice (one) clinics.^¶¶^

**FIGURE:**
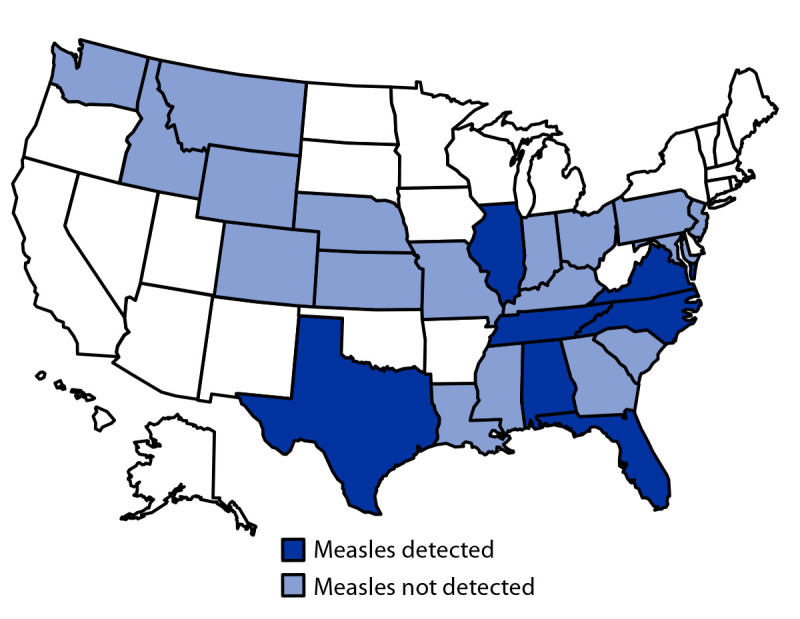
Measles detections using syndromic polymerase chain reaction panel testing (N = 17), by state* — United States, May 2022–April 2023 * Measles virus was detected in panels in the following states: Alabama (three detections), Florida (two), Illinois (one), North Carolina (two), Tennessee (seven), Texas (one), and Virginia (one).

### Characteristics of Persons with Measles Virus Detected by Syndromic PCR Panels

Among 14 children with detectable measles virus for whom laboratory reports were available, the median age was 1 year (range = 1–6 years), and 11 (78.6%) were female. All 14 had received an MMR vaccine dose before specimen collection, including 13 who were vaccinated within 21 days of specimen collection (median = 12 days; range = 8–115 days). One outlier (measles virus detected 115 days after vaccination) occurred in a test on a specimen collected from a child whose measles serology was consistent with immunity (measles immunoglobulin G–positive and immunoglobulin M–negative). Seven of the 14 syndromic PCR panels detected one or more additional viruses, including human herpesvirus type 6 (six), enterovirus (four), human herpesvirus type 7 (three), and Epstein-Barr virus (two). Public health agencies were immediately notified by clinicians about the initial two persons in Tennessee and retrospectively followed up with clinicians about the 12 remaining persons whose positive test results had not been previously reported. All 14 children had been evaluated for rash illness. Fever was reported for at least eight children, including four for whom cough or coryza were also reported, thereby meeting the Council of State and Territorial Epidemiologists’ measles clinical case definition. None of the 14 children had any known epidemiologic risk factors for measles, and no subsequent measles cases were linked to them. For two specimens with test results initially reported to the Tennessee Department of Health, subsequent genetic typing was conducted by CDC. For one, MeVA was inconclusive,*** and for the other, genotyping results were consistent with measles vaccine virus (genotype A).

## Discussion

These measles virus detections by syndromic PCR panels were attributed to previous MMR vaccination because nearly all occurred in persons without risk factors for measles and shortly after receipt of MMR vaccine. Detection of the strain of measles virus used in MMR vaccine typically occurs within 21 days of vaccination, but detection >100 days later has been reported (*7*), a period that aligns with findings described here. Children frequently experience symptoms of rash and fever from many causes, including other viral illnesses and typical vaccine side effects. This investigation identified an alternative viral etiology for rash for one half of the patients with measles virus detections.

Measles is a highly contagious airborne infection that can infect 90% of susceptible contacts (*1*). Preventing measles is essential for population and personal health. Prodromal signs and symptoms include high fever (up to 105°F [40.6°C]) and either cough, coryza, or conjunctivitis. The prodrome is followed by a maculopapular rash that spreads from head to trunk to lower extremities (*8*). Severe complications include pneumonia, encephalitis, and death. Viral transmission can occur from 4 days before to 4 days after rash onset. A single MMR vaccine dose is 93% effective in preventing measles, and receipt of 2 doses is 97% effective (*4*). In the United States, 90% of children receive MMR vaccines by age 24 months (*1*). Although measles is not endemic in the United States, cases and outbreaks occur sporadically when cases are imported from parts of the world where measles remains endemic (*8*). Preventing further measles transmission after detection of a case requires a rapid and robust public health response that can include isolating ill persons, verifying immunity of exposed persons, offering postexposure prophylaxis with measles vaccine or immunoglobulin, and implementation of quarantine measures if necessary (*8*). Notifying public health agencies immediately is imperative to determine which response measures are needed when measles is detected or clinically suspected.

As demonstrated by this analysis, inclusion of measles virus in syndromic PCR panels can result in incidental detection of measles vaccine virus. Some clinicians who received reports of measles detection by syndromic PCR panels anecdotally shared with health departments that they had neither suspected measles infection in the patient nor realized that the test panel included measles. These clinicians had diagnosed common childhood illnesses, such as roseola or impetigo before receiving test results. When choosing diagnostic tests to evaluate skin rash illnesses, clinicians should consider likely etiologies and determine whether laboratory findings will guide treatment recommendations. Syndromic PCR panels provide the opportunity to rapidly test for multiple pathogens, including those unlikely to cause the illness in question. Inability of these testing panels to differentiate between measles virus causing illness and incidental detection of measles vaccine virus RNA can have significant public health reporting and response ramifications, potentially leading to misdiagnosis of measles virus infection. Any detection of measles virus by syndromic PCR testing, even if suspected to be incidental detection of vaccine strain, should be reported to public health agencies immediately so that appropriate investigation and additional testing can proceed if indicated.

In collaboration with CDC, the state health departments that conducted this analysis developed a process to assist public health agencies in determining response measures that consider risk factors and pretest probability of measles infection when measles virus is detected by syndromic PCR panels (Box). Investigators should determine MMR vaccination status and date of receipt and assess whether the person has epidemiologic risk factors for measles. Because signs and symptoms of vaccine reactions can be similar to those associated with measles infection (*9*), clinical presentations consistent with the measles case definition should be interpreted within the context of identified risk factors for measles. If a person was not recently vaccinated, public health response measures to prevent measles virus transmission are necessary and should include specimen referral for genotyping. However, if the person who received the positive test result was vaccinated during the preceding 21 days and has no epidemiologic risk factors (e.g., travel to a region with endemic measles or a known exposure to a person with measles), further public health response is likely unnecessary, because the positive test result likely represents detection of the attenuated vaccine strain measles virus. For persons who were vaccinated within the preceding 21 days and have a risk factor for measles, public health measures to prevent measles transmission should continue while testing for measles vaccine virus by MeVA or genotype. Genetic confirmation of vaccine reaction might also be considered if a person was vaccinated >21 days earlier and has no epidemiologic risk factors.

BOX Proposed public health approach* to incidental detection of measles virus, by syndromic polymerase chain reaction panels

**Public health evaluation**

- Documentation of receipt of measles vaccine
- Evaluation of clinical signs and symptoms^†^
- Assessment of risk factors
  - Epidemiologic link to a measles case^§^
  - Elicitation of travel history^¶^

**Public health response**

- No recent vaccination**
  - Full public health response^††^
- Recent vaccination** with risk factor
  - Full public health response while awaiting confirmatory testing^§§^
- Recent vaccination** without risk factor
  - No further public health response

* Proposed approach developed by CDC and state health departments of Florida, Illinois, North Carolina, Tennessee, Texas, and Virginia.

^†^ Measles infection typically appears as a prodrome of fever with cough, coryza, and conjunctivitis followed by a descending maculopapular rash. Because vaccine reactions can occur with similar symptoms, signs and symptoms consistent with the measles case definition should be interpreted in the context of identified risk factors for measles.

^§^ Including exposure to persons with confirmed measles infection and persons with compatible signs and symptoms.

^¶^ Including travel to areas with measles virus transmission or travel through an international airport.

** Recent vaccination is defined here as receipt of a measles-containing vaccine within the preceding 21 days. If measles virus is detected after 21 days in a vaccinated person without risk factors or signs and symptoms consistent with measles infection, health departments could consider confirmatory testing for vaccine strain to differentiate between wild-type and vaccine strains of measles virus.

^††^ A full public health response includes identifying persons exposed to measles, checking presumptive evidence of immunity, offering postexposure prophylaxis, and recommending isolation or quarantine measures as appropriate to contain the spread of measles.

^§§^ Confirmatory testing by measles vaccine assay real-time reverse transcription–polymerase chain reaction or genotyping is available through the Association of Public Health Laboratories (https://www.aphl.org/aboutAPHL/publications/Documents/VPD-Reference-Guide.pdf) and CDC Vaccine Preventable Diseases Reference Centers (https://www.cdc.gov/measles/lab-tools/genetic-analysis.html).

### Limitations

The findings in this report are subject to at least three limitations. First, 12 persons with measles detected by syndromic PCR panels were not reported to public health agencies, and descriptions of their clinical signs, symptoms, and risk factors are limited to clinician recall and documentation, increasing susceptibility for recall bias. Second, the small number of syndromic PCR panels and measles detections in only a subset of states limits generalizability. Finally, because of delayed reporting to public health officials, only two specimens underwent confirmatory molecular testing.

### Implications for Public Health Practice

During the first year of measles inclusion in commercial syndromic multiplex PCR panels, approximately 1% of tests reported a positive measles test result after recent routine childhood MMR vaccination. These positive test results most likely represented detection of measles vaccine virus in patients with rashes from a vaccine reaction or other cause, rather than measles infection. To facilitate appropriate public health response, clinicians should notify their local public health agency immediately if they are concerned about possible measles infection or patients receive positive measles test results. Commercial laboratories should critically evaluate use of measles in syndromic PCR panels and rapidly notify public health officials of any measles-positive specimens. When measles infection is not clinically suspected but detected by syndromic PCR testing, public health agencies should consider the likelihood of incidental measles vaccine virus detection by assessing measles vaccination history and risk factors. Because 1 dose of MMR vaccine is 93% effective in preventing measles (*4*), if a person recently received MMR vaccine and has no risk factors for acquiring measles, additional public health response is likely unnecessary. However, if a person has not recently received MMR vaccine, subsequent public health response should include necessary measures to prevent measles transmission. For a person who recently received MMR vaccine and has a risk factor for acquiring measles, additional testing for measles vaccine virus is needed to determine subsequent response measures.

## Footnotes

* These authors contributed equally to this report.

† In this report, MMR vaccines refer to both MMR vaccines and measles, mumps, rubella, and varicella vaccines.

§ The state health departments of Florida, Illinois, North Carolina, Tennessee, Texas, and Virginia partnered for this investigation.

¶ Two syndromic panels that use target-enriched multiplex PCR technology available from one commercial laboratory were used for this analysis. Both are marketed for evaluating skin rash illnesses by testing oropharyngeal specimens for *Streptococcus pyogenes* and either seven or nine viruses, including measles.

** According to the Council of State and Territorial Epidemiologists’ measles case definition, measles is an acute illness characterized by a generalized maculopapular rash lasting ≥3 days; temperature of ≥101°F (≥38.3°C); and cough, coryza, or conjunctivitis. https://ndc.services.cdc.gov/case-definitions/measles-2013

†† MeVa is a real-time reverse transcriptase-PCR assay that detects measles vaccine strains.

§§ 45 C.F.R. part 46, 21 C.F.R. part 56; 42 U.S.C. Sect. 241(d); 5 U.S.C. Sect. 552a; 44 U.S.C. Sect. 3501 et seq.

¶¶ The pediatric and family practice clinics also provide walk-in clinic services.

*** The MeVA result was inconclusive, likely because media used by the commercial laboratory is incompatible with MeVA testing and requires genotyping to detect measles vaccine strains.
